# Supplementary material for: Enkephalinergic Neurons in Barrington’s Nucleus Gate Sex-Specific Control of Micturition
Source: Res Sq. 2025 Jul 2:rs.3.rs-6940959. Preprint. [Version 1] doi: 10.21203/rs.3.rs-6940959/v1 (PMC12236902; doi:10.21203/rs.3.rs-6940959/v1)
Supplement: 1 [file NIHPPRS6940959V1-supplement-1.pdf]

Supplementary Items

Cluster Name	Marker Genes	Source	Summary	Included in Further Analyses?
**1_Gm32122-Gulp1**	NA	NA	No Unique or Verifiable Markers (Noncoding RNA or NA)	Excluded
**2_Dach2-Lhx4**	*Dach2, Lhx4*	ABA ISH data	Detected outside of Bar	Excluded
**3_Prlr_Otof**	*Prlr, Otof*	RNAScope ISH	Detected within Bar / In close proximity	Included
**4_Tnc-Zeb2**	*Tnc, Zeb2*	RNAScope ISH, ABA ISH data	Detected outside of Bar	Excluded
**5_Crhbp_Maoa**	*Maoa, Th*	IHC, ABA ISH data	Detected outside of Bar	Excluded
**6_Tac1-Slc17a8**	*Tac1, Slc17a8*	RNAScope ISH	Detected within Bar / In close proximity	Included
**7_Sox6-Cdh6**	*Sox6, Cdh6*	RNAScope ISH	Detected outside of Bar	Excluded
**8_Fign-Sst**	*Fign, Sst*	RNAScope ISH, ABA ISH data	Detected outside of Bar	Excluded
**9_Crh-Reln**	*Crh, Reln, Npas1*	RNAScope ISH, ABA ISH data	Detected within Bar / In close proximity	Included
**10_Penk-Chst9**	*Penk, Tfap2b*	RNAScope ISH	Detected within Bar / In close proximity	Included
**11_Nfib-Nfia**	*Nfib, Nfia*	ABA ISH data	Sparse / Widespread Expression	Excluded
**12_Fgf10-Calcr**	*Fgf10, Calcr, Oprk1*	RNAScope ISH	Detected within Bar / In close proximity	Included
**13_Foxp2-Nps**	*Foxp2, Lhx9*	RNAScope ISH	Detected within Bar / In close proximity	Included
**14_Adamts-Bnc2**	*Bnc2*	RNAScope ISH	Detected outside of Bar	Excluded
**15_Kcna1-Syt2**	*Kcna1, Syt2*	ABA ISH data	Sparse / Widespread Expression	Excluded
**16_Cadps2**	*Cadps2*	ABA ISH data	Detected outside of Bar	Excluded
**18_NA**	NA	NA	No Unique or Verifiable Markers (Noncoding RNA or NA)	Excluded
**17_D030068K23Rik**	NA	NA	No Unique or Verifiable Markers (Noncoding RNA or NA)	Excluded
**19_NA**	NA	NA	No Unique or Verifiable Markers (Noncoding RNA or NA)	Excluded
**20_Gad2-Auts2**	NA	NA	No Unique or Verifiable Markers (Noncoding RNA or NA)	Excluded
**21_Lama1-Inhba**	*Inhba, Grin2c*	RNAScope ISH, ABA ISH data	Detected outside of Bar	Excluded
**22_Slc38a11-Scor2**	*Slc38a11, Dpy19l1*	ABA ISH data	Sparse / Widespread Expression	Excluded

Supplementary Table 1. Marker Genes Used for Spatial Mapping of Putative Bar Populations. *Related to Fig. 1.*
Summary of marker genes used to categorize each of the 22 clusters based on spatial expression patterns, determined using RNA In Situ Hybridization (ISH) or immunohistochemistry (IHC), and/or publicly available ISH image data from the Allen Mouse Brain Atlas (ABA, Allen Institute for Brain Science, 2004). Clusters categorized as "Detected within Bar / In close proximity" were included in further analysis, while those categorized as "Detected outside of Bar" and "Sparse / Widespread Expression," or "No Unique or Verifiable Markers (Noncoding RNA or NA)" were excluded from further consideration. Specific markers for each cluster are provided, and the inclusion or exclusion of a cluster from further analysis is noted.

Sanger sequencing Knock-In alleles primers		
**Prlr_ Primers #**	**Forward_ Sequence (5' -> 3')**	**Reverse_ Sequence (5' -> 3')**
Prlr F3	TGGCCAGCTTTACTGCAACC	
Cre R3		GCTAGAGCCTGTTTTGCACG
Cre F2	AATGGTTTCCCGCAGAACCT	
Cre R6		CAGCGTTTTCGTTCTGCCAA
Cre F3	TTGGCAGAACGAAAACGCTG	
Prlr R4		AGCAGACCACATTACTTCATGACT
**Fgf10_ Primers #**		
11F	TTTAACTGGCAGCACAATGGC	
11R		GCATCCTTCAGCCCCTTGTT
2F	CAACGTCTGTAGCGACCCTT	
2R		AGGTTCTGCGGGAAACCATT
3F	AATGCTTCTGTCCGTTTGCC	
3R		CGGTGCTAACCAGCGTTTTC
4F	TGGCATTTCTGGGGATTGCT	
4R		ACCATTGCCCCTGTTTCACT
14F	GCGCCCTGGAAGGGATTTTT	
14R		TCCATCCAAACTTGACTTGCCT
**Genotyping primers**		
**Prlr_ Primers #**	**Forward Primer**	**Reverse Primer**
Prlr-P2A-Cre (mutant allele); 565bp	(Prlr-F3): TGGCCAGCTTTACTGCAACC	(Cre-R3): GCTAGAGCCTGTTTTGCACG
Prlr-WT; 717bp	(Prlr-F3): TGGCCAGCTTTACTGCAACC	(Prlr-R3): TGCATACAGGCAATGGGTCAT
Generic Cre; 434bp	(Cre-F2): AATGGTTTCCCGCAGAACCT	(Cre-R5): GGTGCTAACCAGCGTTTTCG
**Fgf10_ Primers #**		
Fgf10-IRES-Cre (mutant allele); 1060bp	(Fgf10-F3): GGCAGGCAAATGTATGTGGCATTG	(RVS1 Cre): GCATTGCTGTCACTTGGTCG
Fgf10-WT; 618bp	(Fgf10-F3): GGCAGGCAAATGTATGTGGCATTG	(Fgf10-R2): TTGTGCATATGATATGACCCAAGTGC
Generic Cre; 434bp	Cre-F2): AATGGTTTCCCGCAGAACCT	(Cre-R5): GGTGCTAACCAGCGTTTTCG

Supplementary Table 2. Sanger Sequencing and Genotyping Primers for *Prlr* and *Fgf10* Knock-In Alleles. *Related to Online Methods.*

List of the primer sequences used for Sanger sequencing and genotyping of *Prlr-P2A-Cre* and *Fgf10-IRES-Cre* knock-in alleles. Separate primer sets are provided for mutant and wild-type allele identification, along with generic Cre `primers used for confirmation.

Brain Region	Bregma Levels	All *Penk*⁺		Spinally projecting *Penk*⁺			
		L7PR	D7PN	G56PR	G57PL	L77PL	L78PRL
**Medulla**							
**Gi**, gigantocellular reticular nucleus	*-5.7 to -7.2*	–	4	4	–	8	4
**LPGi**, lateral paragigantocellular nu.	*-5.7 to -7.1*	–	4	2	8	14	2
**MVe**, medial vestibular nucleus	*-5.4 to -6.0*	4	8	8	–	–	–
**Rpa**, raphe pallidus	*-6.4; -7.0*	2	–	2	–	–	–
**IRt**, intermediate reticular nucleus	*-5.4 to -6.4; -7.2 to -7.4*	6	20	4	–	4	2
**SN**, Solitary nucleus	*-6.0 to -6.6;*	–	12	–	4	–	–
**Pons**							
**CG**, central gray	*-5.3*	–	–	–	–	2	2
**Bar,** Barrington's nucleus (contralateral and interconnected)	*-5.3 to -5.5*	4	4	2	4	4	4
**DMTg,** dorsomedial tegmental area	*-4,9; -5.3*	–	–	2	–	2	–
**LDTg,** laterodorsal tegmental nu.	*-5.1 to -5.5*	6	–	4	8	2	2
**LPBn,** lateral parabrachial nucleus	*-5.2*	2	–	–	–	–	2
**MPBn,** medial parabrachial nu.	*-4.9*	–	–	2	–	2	–
**PnC,** pontine reticular nu., caudal	*-4.9 to -5.6*	12	8	2	8	4	–
**PnO,** pontine reticular nu., oral	*-4.3 to -4.8*	8	–	12	8	8	2
**PPN**, pedunculopontine nucleus	*-4.6 to -5.0*	2	4	8	12	8	8
**Su5,** supratrigeminal nucleus	*-5.1*	2	–	2	–	–	–
**SubC,** subcoeruleus nuclei	*-5.0 to -5.3*	4	12	16	8	6	4
**Midbrain**							
**dmPAG,** dorsomedial peri-aqueductal gray	*-3.9 to -4.9*	2	–	6	4	6	2
**lPAG,** lateral periaqueductal gray	*-4.0 to -4.9*	4	4	24	–	12	24
**mRt,** mesencephalic reticular formation	*-3.3 to -3.8; -4.3 to -4.7*	6	16	2	12	6	4
**PMnR,** paramedian raphe nucleus	*-4.4 to -4.8*	–	–	2	–	–	4
**SCs,** Superior colliculus	*-3.5 to -3.8; -4.3 to -4.7*	6	12	4	8	4	2
**vlPAG,** ventrolateral periaqueductal gray	*-4.3 to -5.1*	20	16	64	20	50	44
**Hypothalamus**							
**DMH,** dorsomedial hypothalamic nu.	*-2.0 to -2.3*	8	–	–	4	–	–
**LH,** lateral hypothalamic area	*-1.2 to -2.5*	10	16	24	44	10	24
**LPOA,** lateral preoptic area	*0.0 to -0.3*	4	4	4	4	–	10
**MPA,** medial preoptic area	*0.1 to -0.2*	–	–	2	8	4	12
**PH,** posterior hypothalamic nucleus	*-2.1 to -2.5*	2	–	6	4	2	6
**SubI,** subinsertal nucleus	*-1.3 to -1.6*	–	4	18	24	10	14
**VMH,** ventromedial hypothalamic nu.	*-1.7 to -2.0*	2	4	2	8	4	12
**Zi,** zona inserta (caudal part)	*-2.5 to -2.8*	4	4	–	–	10	–
**Cerebral Nuclei**							
**BST,** bed nu. of the stria terminalis	*0.2 to -0.1*	24	28	6	4	–	–
**CeA,** central amygdaloid nucleus	*-1.2 to -1.7*	40	40	10	–	4	4

Supplementary Table 3. Brain input sites to Bar*Penk* neurons. *Related to Fig. 8.*

Number of rabies-transfected neurons detected across 33 putative input sites to all *Penk*-expressing neurons in Bar (n = 2) and spinally projecting Bar^{Penk} neurons (n = 4). Input sites are categorized by macrostructures, including the medulla, pons, midbrain, hypothalamus, and cerebral nuclei. The second column provides the approximate Bregma span of eGFP-labeled cells.

Supplementary Video 4. 3D Visualization of Upstream Inputs to Bar^{Penk}. *Related to Fig. 8.*
A 3D reconstruction of the whole brain, cleared and stained using the iDISCO+ protocol, showing BarPenk starter cells (red spheres) and RVdG-labeled upstream neurons (green spheres). The video first displays a 360° rotation from a lateral view, followed by a walkthrough in coronal orientation progressing caudal to rostral. Tissue autofluorescence provides structural context. Scale bar shown in lower left corner.
